# Impact of Undergraduate Research as a Compulsory Course in the Dentistry Study Program Universitas Indonesia

**DOI:** 10.3390/dj10110204

**Published:** 2022-10-28

**Authors:** Lisa R. Amir, Sri Angky Soekanto, Vera Julia, Nieka A. Wahono, Diah Ayu Maharani

**Affiliations:** 1 Department of Oral Biology, Faculty of Dentistry, Universitas Indonesia, Jakarta 10430, Indonesia; 2 Dental Education Unit, Faculty of Dentistry, Universitas Indonesia, Jakarta 10430, Indonesia; 3 Department of Oral and Maxillofacial Surgery, Faculty of Dentistry, Universitas Indonesia, Jakarta 10430, Indonesia; 4 Department of Pediatric Dentistry, Faculty of Dentistry, Universitas Indonesia, Jakarta 10430, Indonesia; 5 Department of Preventive and Public Health Dentistry, Faculty of Dentistry, Universitas Indonesia, Jakarta 10430, Indonesia

**Keywords:** undergraduate research, dental education curriculum, evaluation

## Abstract

Undergraduate (UG) research is regarded as a fundamental component in dental education. The present study was designed to examine the perception of the clinical students and the graduates of dentistry profession programs in the past 10 years on UG research as a compulsory course at the Faculty of Dentistry Universitas Indonesia. A total of 310 respondents, consisting of clinical students (64.8%) and alumni (35.2%), participated in this study. The majority of respondents (81.3%) agreed to UG research as part of compulsory courses in the curriculum of dentistry study programs. The positive impact of UG research on their professional careers was perceived by 78.3% of participants. Only 11.6% of participants responded that UG research experiments were not important in dental education, and 18.7% preferred UG research as an elective course. UG research as a compulsory course in the dental curriculum was well received by the majority of participants. Recommendations included student autonomy to select research topics of interest, longer duration to complete UG research, and more opportunities to present the research results in scientific conferences and to publish in scientific journals. Dental schools and their faculties play essential roles in improving the research environment for undergraduate dental students.

## 1. Introduction

The implementation of scientific research and scholarly activity in dental education has become crucial to sustain the advancement of oral health and to support the creation of lifelong learners. Higher education recognizes the importance of research training to produce scientifically oriented practitioners. The incorporation of research requirements as part of undergraduate curriculums has been recommended in the global congress on dental education in 2007 [1]. There is growing recognition that the research and technological bases of dentistry and other health professions are rapidly changing. Dental schools need to equip future dentists to continue to develop and to understand the processes of research; to critically assess or report on new materials and methods of innovation in dentistry [2]. This must be supported by a curriculum that promotes science and innovation, incorporates the knowledge of technological advances, and evidence-based approaches [3,4,5,6,7].

The benefits of scientific research training for students have been reported [4,8,9,10,11]. Stronger cognitive personal skills and higher satisfaction with their educational experiences were observed in a group of students with undergraduate research experience [5,7]. Other studies have demonstrated the positive impact on both career development and academic success, helping to develop the scientific mindset [4,12]. Numerous dental higher education institutions throughout the world have introduced various ways of exposing students to research [2,3,4,5,6,7,11,12]. Undergraduate research opportunities have been offered as a summer research program of extracurricular activity [13,14], elective courses for interested students [5,12,13,14,15], or compulsory courses necessary for graduation [2,6,11]. Dental undergraduate research programs are available in dental schools in China, although participation is not aimed to gain credits, as the research projects are undertaken as complementary assignments. The program is designed to improve innovative learning and critical thinking during dental professional training sessions. The study concluded the key essential elements for the success of undergraduate research programs involved the support of dental schools, the engagement of qualified faculty members, and the interest and autonomy of students [5].

Undergraduate research has been part of a compulsory course in the dental curriculum by the Faculty of Dentistry, Universitas Indonesia, since 2005. The course is intended to give opportunities to students to develop independent and critical thinking, and enhance a scientific mindset. During the sixth and seventh semesters of the academic undergraduate (BSc Dent) program, students conduct research under the supervision of faculty members, to assist them in completing their projects. The majority of undergraduate research was associated with a lecturer’s research project, and funded by intra- or extramural research grants. Students prepare research proposals, conduct the study, and report the research results as their bachelor theses after approximately 1 year. However, the impact of research as a compulsory course in the undergraduate dentistry study program is still unclear. The present study was designed to examine the experience of implementing undergraduate research programs as a compulsory course in our dental curriculum. Specifically, we aimed to analyze the following: [1] student and alumni perceptions of their undergraduate research experiences and overall satisfaction; [2] and its impact on both career development and academic success. We hypothesized the undergraduate research experiments had a positive impact on students.

## 2. Materials and Methods

### 2.1. Sampling Procedures and Participants

This study was performed from March 2022 to July 2022. Study participants were clinical students of professional programs and alumni of the Faculty of Dentistry, Universitas Indonesia, during the last 10 years. All participants received a dental curriculum in which undergraduate research was part of a compulsory course. However, during the COVID-19 pandemic, as limited access to the research facilities was applied, students were given the option to perform systematic reviews as their bachelor theses; these were excluded in this study. Convenience sampling methods were used in this study. Prior to filling out an online questionnaire, all participants gave consent to participate in this study. Ethical approval of this study was obtained from the Dental Research Ethics Committee, Universitas Indonesia (17/Ethical Approval /FKGUI/IV/2022).

### 2.2. Undergraduate Research Course in Dental Curriculum

Undergraduate (UG) research was initially included in the dental curriculum as an elective course. Students could choose to perform UG research or write a literature review paper as their bachelor theses. UG research was later introduced in the 2005 dental curriculum as a compulsory course. Students are required to perform research in one of the assigned departments in the faculty, which consist of: Conservative Dentistry, Dental Materials, Oral Biology, Oral Medicine, Oral Radiology, Oral Surgery, Orthodontics, Pediatric Dentistry, Periodontics, Prosthodontics, or Public Health.

The UG research course is delivered in Semester 7, while courses that support UG research, such as research methodology and biostatistics courses, are delivered in Semesters 5 and 6, respectively. The courses provide a comprehensive introduction to research methodology, research proposal writing, and statistical analysis to support the undergraduate research course. Student-centered learning is implemented to foster integrated approaches to learning, which include interactive lectures, self-directed learning, collaborative learning discussions, and individual and group assignments. Students prepare research proposals as their final assignment for the research methodology course. This course is designed to help students develop the skills to pose novel research questions and formulate scientific frameworks.

Two supervisors are assigned to support student research. Research topics are first discussed with the supervisors. Students can propose their own research ideas; however, the majority of UG research is associated with the supervisor’s ongoing research projects that have received research funding. Under the guidance of their research supervisor, students conduct a comprehensive literature review of the topic of interest, generate hypotheses, formulate conceptual frameworks, research designs, gather/collect data, conduct data analyses and interpretation, and write the research report as their bachelor thesis. It is not mandatory to write a manuscript for publication.

Over the past two years, students’ preferences for the departments in which they are interested in performing their research have also been taken into account. Another consideration is the quota of student intake. In general, preclinical departments allow higher student intake for UG research compared to clinical departments.

### 2.3. Questionnaire

Developed using Google, an online questionnaire was created to assess the participant’s perception of their UG research program as one of the compulsory courses in the dental curriculum. The questions were divided into general information, consisting of 16 questions, and respondent’s perception related to their preference, learning satisfaction, learning experiences, and the impact of UG research on their academic performances and professional careers, consisting of 17 questions. The response options represented 4 Likert-type scales (0 = strongly disagree to 3 = strongly agree). The last open-ended question provided respondent’s general feedback on UG research in the dental curriculum. There were 34 questions overall. The questionnaire was designed to have positive and negative questions to reduce the acquiescence bias. The questionnaire was drafted and agreed by an expert panel formed to ensure the validity of the items. The face validity of the questionnaire was assessed by fifteen students who were randomly chosen to obtain a clear picture of the clarity and comprehensiveness of the questionnaire. The survey was anonymous and distributed through the WhatsApp communication tool and email. Three student council members helped with the recruitment of study participants to avoid conflict of interests.

### 2.4. Statistical Analysis

Data distribution was examined by using Kolmogorov–Smirnov test. The internal consistency reliability questionnaire measured by Cronbach’s alpha was 0.908. Descriptive statistics were computed and bivariate analyses were performed. The level of significance was set as *p* < 0.05.

## 3. Results

### 3.1. General Information

The questionnaire was distributed to clinical students and alumni of the dentistry program delivered by the Faculty of Dentistry, Universitas Indonesia, from the last 10 years. A total of 310 participants returned the questionnaire, consisting of clinical students of the dentistry program delivered by the Faculty of Dentistry, Universitas Indonesia (64.8%), and graduates from the past 10 years (35.2%) participated in this study. The highest degree among alumni was Doctor of Dental Surgeon (31.3%), although at the time of this study, 38.1% of alumni were postgraduate students (master, PhD, or specialization program students) (Table 1). Cronbach’s alpha for the questionnaire was 0.908. No Corrected Item Total Correlation (CITC) value was lower than 0.30, which allowed all items to be included in the instrument.

### 3.2. Undergraduate Research Experiences

More than half of respondents conducted laboratory studies for their UG research, followed by epidemiological studies. Clinical studies were performed the least in UG research due to a limited quota being available in the clinical departments (Table 2). The reports of the UG research results were mainly stored in the Universitas Indonesia library repository, while 38.7% of the research reports have been published or were in preparation to be published in national/international journals. Less than 20% of participants experienced presenting their UG research in scientific conferences or participated in scientific competitions (Table 2).

### 3.3. Perception of Undergraduate Research

In terms of preference, the majority of respondents agreed and strongly agreed with UG research is important as part of compulsory courses in the curriculum of the dentistry study program. Only 11.6% of participants responded that UG research experiments were not important in dental education, and 18.7% disagreed with UG research being one of the compulsory courses in the curriculum. Based on the response to the open-ended questions, factors that influenced these perceptions included the support received from research supervisors and the opportunities to choose the research topics based on student interests (Table 3). Adequate support from research supervisors helped students to finish the UG research course in a timely manner. Furthermore, having UG research results published in the scientific journal influenced the student perception of the UG research experience. Students that published their research reports had more significantly positive UG research experiences (Table 4).

In the learning experiences domain, more than 90% of participants agreed that UG research improved their problem-solving skills, independent and critical thinking skills, and their ability to deliver presentations. A total of 12% of participants disagreed that UG research taught them the relationship between research and clinical practice (Table 3).

In the learning satisfaction domain, 12.2% of participants were not satisfied with their UG research experience. Based on the response of the open-ended question, the reasons for dissatisfaction were the short duration time to complete the UG research course and the research topics that might not have aligned with students’ interests, although they agreed having UG research experiences was beneficial. The majority agreed there were adequate research facilities and support from research supervisors.

### 3.4. Impact of Undergraduate Research

The results showed a positive impact of UG research on professional careers, motivating students to pursue academic careers and to continue postgraduate education; more than 50% of participants agreed and strongly agreed with the impact of UG research (Table 5).

## 4. Discussion

Undergraduate (UG) research is regarded as a fundamental component in dental education for the academic development of dental students. As such, compulsory course-based undergraduate research has been implemented in the curriculum of numerous dental schools [2,6,11]. UG research requires a lot of time, effort, and funding; therefore, the impact of this research experience on educational outcomes needs to be evaluated. Student feedback could provide important information for the evaluation of UG research, necessary for the improvement of a new curriculum. The present study assessed the student and alumni’s perspective of UG research experience that was mandatory for graduation. The overall feedback was very positive and well received. Over three quarters of responses ranged from “agree” to “strongly agree” for UG research as a compulsory course in the dental curriculum. The BSc Dent program attempts to create a UG research experience, to provide students with the ability to understand the basics of research, including survey design and statistics. It contributes to new knowledge acquisition, to strengthen confidence, and to the development of critical thinking skills that are essential in evidence-based practice. The research experience could inspire students to become lifelong learners [11,14].

In this study, 11.6% of participants responded that UG research experiences were not important in dental education, and 18.7% preferred UG research as an elective course. These negative experiences toward the compulsory UG research course were related to research projects experiences that did not meet participants’ expectations, whereby the UG research topics were mainly part of the supervisor’s research that had received research funding. Determining how UG research experiences can meet the needs of students’ interests, while at the same-time being cost effective, is a significant challenge. The freedom to choose research topics based on students’ interests can have a positive impact on student learning experiences [2]. When students are given the opportunity to make choices in line with their interests, it motivates them to learn and obtain a deeper understanding compared to students who are less interested in the topics [5,16,17]. Lecturers may integrate their research into education to increase student awareness of current research available, and thus, implement research-based education [17]. Therefore, recommendations include an open recruitment system of research assistants for the available research projects, so students can explore their project of interest as part of the compulsory course.

The need to incorporate research into dental education has grown alongside the advancement of scientific knowledge. There have been changes in the attitudes of both the faculty and students with regards to new sciences in dental education. However, a lack of adequate time in the dental curriculum was one of the most frequent barriers to UG research, and was faced by many students. This challenge was also observed in our study and by others [3,11,14]. As students need to simultaneously take other compulsory courses while performing UG research, many of the dental students were in a rush to complete research projects and so wrote bachelor theses while not having sufficient time to extensively learn the background of a particular subject. This resulted in an inadequate understanding of their research projects. Time constraints in dental schools made research less accessible to students without prior experience [3,11,14]. A survey conducted by the American Association for Dental Research National Student Research Group revealed that U.S. dental students strongly agreed that scientific research enabled their progress in dentistry [3]. Inadequate time in the curriculum to perform research was one of the major obstacles reported. Therefore, recommendations include introducing students to the prospective research projects at an earlier time, to give additional time for students to thoroughly learn the background of the study.

Research supervisors play an important role in helping undergraduates to simultaneously deepen their understanding of science and guide them to develop a scientific identity [9,18,19,20,21,22,23,24,25,26]. Ideally, research supervisors guide undergraduates to understand the conceptual knowledge and background information of the research topics, and to develop science practices such as building an argument from evidence. Students learn how to set up experiments and develop data collection skills, but they might have difficulties in understanding the rationale of the experimental design, interpreting results, or relating analyses to research questions. It is the responsibility of the faculty to assist students in understanding the connections between experiences in experimental design, data collection, their own interpretations, and scientific communication [21,22,23,24]. Students often experience obstacles during research and require guidance to overcome problems. These challenges are intensified when students have trouble finding time to discuss their projects with their supervisors, or cannot communicate with them. This can cause frustration and confusion, and could result in an overall negative experience [14].

Evaluating mentoring interaction is crucial to ensure research experiences meet the needs of a diverse range of students. A previous study on mentoring in professional and educational contexts reported that students’ confidence in performing science practice was correlated with the adequate support from the faculty and the frequency of meetings [25]. Moreover, students who received support from the faculty were more likely to go to graduate schools [26]. Our finding showed the majority of supervisors were lecturers with PhD degrees, followed by professors. Students feel relatively supported by their supervisors. Despite the lack of time to perform UG research, the quality of supervision was more than adequate. Previous research on the quality of postgraduate dental research for trainees suggests that communication can be improved by supervisors actively checking-up on students regarding their progress; other suggestions include using forms or keeping notes to track a student’s progress [27]. Other literature regarding UG research highlights the supervisor’s accountability and interests, in order to maintain the student’s interests and accountability [28]. When there is good interaction between research supervisors and students, it allows for the regular monitoring of students’ performance and progress [7].

The present study suggests that the main contributing factors of a good UG research experience demands adequate time for completion and support from the faculty. Research programs benefited students, the faculty, and the dental schools involved. Through research experience, students learn valuable skills in reasoning, problem-solving, and critical thinking. The faculty benefited from increased scholarly activity and assistance in research projects. Dental schools with active research programs might gain greater exposure through scholarly activity and scientific publications. Our findings present evidence of the positive impact of undergraduate research as a compulsory course in the dental curriculum of the dentistry study program. However, there are several limitations of the study, which included a survey distributed to students in the dental profession program and alumni from the past 10 years from one dental school, and achieved only a 24.9% response rate. Therefore, the non-response error 2 cannot be completely ruled out, due to this low response rate. Whether similar responses and challenges are found in other dental schools warrants further investigation.

## 5. Conclusions

The results indicate that having UG research as a compulsory course in the dental curriculum has been well received, though with notable room for improvement. The recommendation for the institution for the improvement of the dental curriculum includes the policy of student autonomy to select research topics of interest, provide longer duration to perform UG research, create an annual research day for UG students, and provide more opportunities to have experiences participating in scientific conferences. Recommendations for the faculty and UG research supervisors are to provide more opportunities for students to get involved in writing manuscripts for publication in the scientific journals. This would not only simultaneously benefit students when they want to continue to pursue postgraduate education, but also the tenure-track and tenured faculty for their productivity obligations in terms of scientific publication. Dental schools and faculties play essential roles in improving the research environment for undergraduate students. It is imperative for dental schools to make the necessary adjustments to meet students’ expectations.

## Figures and Tables

**Table 1:** General Information.

		Total (%)	Clinical Students (Profession Program)	Alumni (<10 Years)
1	Gender			
	Male	63 (20.3%)	35 (17.7%)	28 (25%)
	Female	247 (79.7%)	163 (82.3%)	84 (75%)
2	Average GPA	3.52 ± 0.19	3.55 ± 0.18	3.47 ± 0.20
3	Latest Education			
	Bachelor of Dental Science	200 (64.5%)	200 (64.5%)	-
	Doctor of Dental Surgeon	97 (31.3%)	-	97 (31.3%)
	Master Degree	5 (1.6%)	-	5 (1.6%)
	Specialist Degree	7 (2.3%)	-	7 (2.3%)
	PhD Degree	1 (0.3%)	-	1 (0.3%)
4	Current Position			
	Students of Dental Profession program	200 (64.5%)	200 (64.5%)	-
	Students of Master program	3 (1.0%)	-	3 (1.0%)
	Students of Specialization program	31 (10.0%)	-	31 (10.0%)
	Students of Doctoral program	3 (1.0%)	-	3 (1.0%)
	General Dentists	66 (21.3%)	-	66 (21.3%)
	University Lecturers	5 (1.6%)	-	5 (1.6%)
	Others	4 (1.3%)	-	4 (1.3%)

Most of the respondents were female, which reflects the majority of dental students in our university. Note: The respondents were mainly clinical students who had experienced undergraduate research during the last three years.

**Table 2:** Background of Undergraduate Research.

		Total (%)	Clinical Students (Profession Program)	Alumni (<10 Years)
1	Undergraduate Research was beneficial for my academic success and professional career			
	Yes	281 (90.6%)	186 (66.2%)	95 (33.8%)
	No	29 (9.4%)	12 (41.4%)	17 (58.6%)
2	Research topics			
	Laboratory Studies	173 (55.8%)	106 (61.3%)	67 (38.7%)
	Epidemiological Studies	78 (25.2%)	55 (70.5%)	23 (29.5%)
	Clinical Studies	59 (19.0%)	37 (62.7%)	22 (37.3%)
3	Research Supervisors			
	Professors	73 (23.5%)	46 (63.0%)	27 (37.0%)
	Lecturers (PhD)	157 (50.6%)	101 (64.3%)	56 (35.7%)
	Lectures (Master degree)	38 (12.3%	21 (55.3%)	17 (44.7%
	Lectures (specialization degree)	42 (13.5%)	30 (71.4%)	12 (28.6%)
4	Presentation in scientific conferences			
	International Conferences	31 (10.0%)	12 (38.7%)	19 (61.3%)
	National Conferences	8 (2.6%)	-	(100%)
	Local conference	9 (2.9%)	1 (11.1%)	8 (88.9%)
	No experience	262 (84.5%)	185 (70.6%)	77 (29.4%)
5	Participation in scientific competition			
	International event	27 (8.7%)	12 (44.4%)	15 (56.6%)
	National event	6 (1.9%)	2 (33.3%)	4 (66.7%)
	Local event	0 (0%)	0 (0%)	0 (0%)
	No experience	277 (89.4%)	184 (66.4%)	93 (33.65%)
6	Publication of UG research			
	Published in International journals	40 (12.9%)	16	24
	Published in National journals	16 (5.2%)	6	10
	Manuscript in preparation	54 (17.4%)	51 (94.4%)	3 (5.6%)
	Store in UI repositories	190 (61.3%)	116 (61.1%)	74 (38.9%)

The allocation of research topics and the lecturers assigned as research supervisors were determined by the institution.

**Table 3:** Perception of UG Research Experiences.

Statements on Undergraduate (UG) Research	Likert Score				
	Strongly Disagree	Disagree	Agree	Strongly Agree	Domain Mean ± SD
**A. Preference Domain**					**1.93 ± 0.96**
1. UG Research is not important for academic dental education	118 (38.1%)	156 (50.3%)	2 (0.6%)	34 (11.0%)	
2. UG Research preferably should not be included as a compulsory course in dental curriculum	106 (34.2%)	146 (47.1%)	6 (1.9%)	52 (16.8%)	
**B. Learning Experiences Domain**					**3.54 ± 0.62**
3. UG Research improved my problem solving skills	0 (0%)	13 (4.2%)	105 (33.9%)	192 (61.9%)	
4. UG Research taught me to think independently	0 (0%)	8 (2.6%)	108 (34.8%)	194 (62.6%)	
5. UG Research improved critical thinking skills	0 (0%)	8 (2.6%)	127 (41.0%)	175 (56.5%)	
6. Improve my ability to deliver presentation in seminars	4 (1.3%)	28 (9.0%)	88 (28.4%)	190 (61.3%)	
7. UG Research thought me the relation of research and clinical practice	1 (0.3%)	36 (11.6%)	77 (24.8%)	196 (63.2%)	
**C. Learning Satisfaction Domain**					**3.16 ± 1.02**
8. UG Research experience during my academic study was not satisfactory	91 (29.4%)	181 (58.4%)	5 (1.6%)	33 (10.6%)	
9. UG Research experience was quite satisfactory	3 (1.0%)	21 (6.8%)	49 (15.8%)	237 (76.5%)	
10. UG Research experience was supported by adequate research facilities	6 (1.9%)	35 (11.3%)	61 (19.7%)	208 (67.1%)	
11. UG Research experience was adequately supported by supervisors	2 (0.6%)	17 (5.5%)	117 (37.7%)	174 (56.1%)	

Note: In the learning experiences domain, participants mainly disagreed that UG research taught them the relationships between research and clinical practice, as more than half of the research topics were laboratory studies. The bold used to highlight different domains tested in this study.

**Table 4:** Researchers Profile with Negative Undergraduate Research Experience.

Variables	UG Research Experience during My Academic Study Was Not Satisfactory		UG Research Is Not Important for Academic Dental Education		UG Research Preferably Not Included as Compulsory Course in Dental Curriculum	
	Score	*p*	Score	*p*	Score	*p*
Gender						
Male	3.1 (0.4)	0.062	3.2 (0.8)	0.953	3.1 (0.9)	0.915
Female	3.1 (0.4)		3.3 (0.7)		3.1 (0.7)	
Latest education						
Bacherlor (DDS)	3.1 (0.7)	0.452	3.2 (0.7)	0.012 *	3.0 (0.8)	0.001 *
Master, PhD	3.2 (0.7)		3.4 (0.7)		3.3 (0.7)	
Current status						
Student of dental profession program	3.1 (0.7)	0.743	3.2 (0.7)	0.017 *	3.0 (0.8)	0.002 *
Others	3.2 (0.7)		3.4 (0.7)		3.3 (0.7)	
Research topics						
Laboratory studies	3.1 (0.7)	0.433	3.3 (0.7)	0.360	3.2 (0.8)	0.400
Epidemiological studies	3.1 (0.7)		3.3 (0.7)		3.1 (0.7)	
Clinical studies	3.2 (0.6)		3.2 (0.6)		3.0 (0.9)	
Research Supervisors						
Professors	3.2 (0.6)	0.484	3.2 (0.6)	0.440	3.0 (0.7)	0.610
Lecturers (PhD)	3.1 (0.7)		3.3 (0.7)		3.2 (0.8)	
Lecturers (Master)	3.1 (0.8)		3.1 (0.7)		3.1 (0.7)	
Lecturers (Clinical Master)	3.1 (0.7)		3.2 (0.7)		3.1 (0.8)	
Presentation in scientific conferences						
No	3.1 (0.7)	0.231	3.2 (0.7)	0.023 *	3.1 (0.8)	0.017 *
Yes	3.2 (0.8)		3.4 (0.7)		3.3 (0.7)	
Participation in scientific competition						
No	3.1 (0.7)	0.241	3.2 (0.7)	0.022 *	3.1 (0.8)	0.130
Yes	3.2 (0.8)		3.5 (0.6)		3.3 (0.8)	
Publication of UG research						
No	3.1 (0.7)	0.005 *	3.2 (0.7)	0.032 *	3.1 (0.8)	0.046 *
Yes	3.3 (0.7)		3.3 (0.7)		3.2 (0.7)	

Note: Participant’s experiences in having their research results published in scientific journals determined their satisfaction, their perception of the importance of UG research, and their preference of UG research as a compulsory course in the dental curriculum. * statistically significant differences were observed between the group tested. The bold used to highlight different domains tested in this study.

**Table 5:** Impact of UG Research.

Statements on Undergraduate (UG) Research	Likert Score				
	Strongly Disagree	Disagree	Agree	Strongly Agree	Domain Mean Preference ± SD
**D. Impact of UG Research**					**3.32 ± 0.87**
1. Helps me to apply my theoretical knowledge and clinical practice	5 (1.6%)	46 (14.8)	59 (19.0%)	200 (64.5%)	
2. UG Research stimulates me to pursue academic careers	9 (2.9%)	83 (26.8%)	64 (20.6%)	154 (49.7%)	
3. Helps me to reflect on technological advancement of new dental materials in clinical practice	8 (2.6%)	49 (15.8%)	82 (26.5%)	171 (55.2%)	
4. Positive impact on my professional career	5 (1.6%)	62 (20.0%)	55 (17.7%)	188 (60.6%)	
5. Positive impact on my current post-graduate program	5 (1.6%)	57 (18.4%)	67 (21.6%)	181 (58.4%)	
6. Motivates me to continue postgraduate education	7 (2.3%)	86 (27.7%)	64 (20.6%)	153 (49.4%)	

The positive impact of UG research was perceived by more than two-thirds of participants. The bold used to highlight different domains tested in this study.

## Data Availability

All of the relevant raw data of this study will be available from Lisa Amir (corresponding author) for any author who wishes to collaborate with us.
